# Patterns of genomic variation in Coho salmon following reintroduction to the interior Columbia River

**DOI:** 10.1002/ece3.3492

**Published:** 2017-10-30

**Authors:** Nathan R. Campbell, Cory Kamphaus, Keely Murdoch, Shawn R. Narum

**Affiliations:** ^1^ Columbia River Inter‐Tribal Fish Commission Hagerman ID USA; ^2^ Yakama Nation Fisheries Yakima WA USA

**Keywords:** Coho salmon, genetic adaptation, genetic monitoring, RAD sequencing, reintroduction

## Abstract

Coho salmon were extirpated in the mid‐20th century from the interior reaches of the Columbia River but were reintroduced with relatively abundant source stocks from the lower Columbia River near the Pacific coast. Reintroduction of Coho salmon to the interior Columbia River (Wenatchee River) using lower river stocks placed selective pressures on the new colonizers due to substantial differences with their original habitat such as migration distance and navigation of six additional hydropower dams. We used restriction site‐associated DNA sequencing (RAD‐seq) to genotype 5,392 SNPs in reintroduced Coho salmon in the Wenatchee River over four generations to test for signals of temporal structure and adaptive variation. Temporal genetic structure among the three broodlines of reintroduced fish was evident among the initial return years (2000, 2001, and 2002) and their descendants, which indicated levels of reproductive isolation among broodlines. Signals of adaptive variation were detected from multiple outlier tests and identified candidate genes for further study. This study illustrated that genetic variation and structure of reintroduced populations are likely to reflect source stocks for multiple generations but may shift over time once established in nature.

## 1 INTRODUCTION

Reintroduction of animals into habitats they once populated is a conservation practice that is being utilized with increasing frequency (e.g., Seddon, Armstrong, & Maloney, 2007). As human influences on habitats have been the root cause of decline, extirpation, or extinction of various species (Dobson et al., 2006; Estes et al., 2011), changes in management policies such as environmental regulations, harvest restrictions, limitations on development, and habitat restoration have made reintroduction a possibility for some species (Corlett, 2015; Wallace, Clark, & Reading, 2002). Many reintroduction efforts, however, fail in their objective to establish viable naturally reproducing populations (Griffith, Scott, Carpenter, & Reed, 1989). The success or failure of reintroduction efforts is dependent on multiple factors including suitability of habitat to support reintroduced species, proper management following reintroduction, and the ability of reintroduced populations to adapt to new habitat (Seddon et al., 2007). Genetic diversity within reintroduced populations provides capacity for adaptive changes to the new environment as beneficial combinations of gene variants become enriched in subsequent generations. Therefore, factors which would reduce genetic diversity such as a small initial reintroduced population (founder effect), high mortality rates following reintroduction (genetic bottleneck), or the breeding of related animals (inbreeding) are taken into careful consideration when planning and managing reintroduction efforts (Haig, Ballou, & Derrickson, 2014; Jamieson, 2011).

Anadromous fishes such as salmonids utilize various habitats throughout their life cycles (small streams, large rivers, estuaries, and the ocean), and alterations to any of them may lead to population declines or extirpation (Gustafson et al., 2007). However, reintroduction efforts for salmon typically focus on spawning habitat as homing behavior (philopatry) is the basis for population structure in these species (Hendry, Castric, Kinnison, & Quinn, 2004; Keefer & Caudill, 2014). In order to establish a new population, juvenile salmon must imprint on a new stream before they begin their migration to the ocean in order to return to the same location as an adult (Quinn 1993; Lohmann, Putman, & Lohmann, 2008; Johnstone, Lubieniecki, Koop, & Davidson, 2011). High mortality rates prior to adulthood can mean replacement levels may not be met for newly established naturally spawning salmon populations. For these reasons, many populations of salmon are kept viable using hatchery supplementation to produce enough juvenile fish to maintain adult returns at or above replacement (Fast et al., 2015; Ford, Murdoch, Hughes, Seamons, & LaHood, 2016; Hess et al., 2012).

In the Columbia River drainage in the Pacific Northwest of the United States, Coho salmon historically utilized many of the upriver tributaries as viable spawning habitat. However, as with other salmonid species, large portions of Coho salmon spawning habitat were either blocked by impassable dams or otherwise rendered unusable by this species. Early mitigation efforts to restore salmonid species through hatchery programs in the Columbia River system focused mainly on Chinook salmon, Sockeye salmon, and steelhead, while little effort was made toward restoring Coho salmon populations (Kareiva, Marvier, & Mcclure, 2000; Paquet et al., 2011; Williams, 2008). As a result, by the 1980s, all Coho salmon populations above Bonneville Dam (river mile 145) had been extirpated (Galbreath, Bisbee, Dompier, Kamphaus, & Newsome, 2014). In the late 1990s and early 2000s, the Yakama, Umatilla, and Nez Perce tribes began hatchery‐based reintroduction programs in several mid and upper Columbia River tributaries (Yakima R., Wenatchee R., Methow R., and Clearwater R.) using offspring from lower Columbia River adult fish as their source stock.

In the Wenatchee River, Coho salmon from lower Columbia River stocks were reintroduced in order to establish a locally adapted population (Bosch et al., 2007; Galbreath et al., 2014). Releases of juvenile smolts (1.5 year old) in 1999, 2000, and 2001 produced the first generation of 3‐year‐old adult returns in 2000, 2001, and 2002 (Galbreath et al., 2014; Murdoch, Prevatte, & Kamphaus, 2005). Although first‐generation fish in 2000 and 2002 required supplementation with lower Columbia River juveniles in order to meet production goals, the program quickly transitioned to exclusively using fish returning to the Wenatchee system as broodstock. Selective forces acting on the new population were especially significant due to freshwater migration distances more than 10 times that of their source stock for both emigrating smolts and returning adult fish (Wenatchee River [756 km migration]; source stock [70 km migration]). Phenotypic differences correlating with freshwater migration distances in anadromous fish have been previously reported (Crossin et al., 2004; Jonsson & Jonsson, 2006). Energetic demands and exposure to stress related to increased migration distance are hypothesized to create selective pressure on the new Coho salmon population which may manifest as significant population‐wide changes in allele frequencies within areas of the genome.

In this study, we examine patterns of neutral and adaptive genetic variation in this reintroduced population of Coho salmon over the course of four generations. To screen for signals of genetic adaptation, SNP markers throughout the genome were examined for significant changes in allele frequency over multiple generations to identify outlier loci that exhibited evidence for divergent selection as opposed to genetic drift. As Coho salmon populations can display distinct temporal structure following three‐year age classes (Smith et al., 2015), this provided the opportunity to test for consistent outlier results among biological replicates within the same study system.

## 2 METHODS

### 2.1 Tissue samples

**Figure 1:**
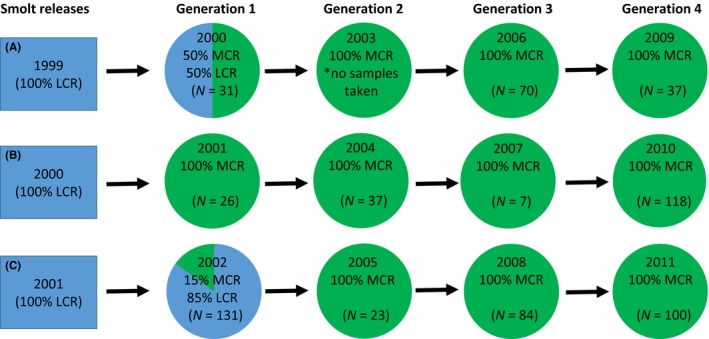
Coho broodstock samples. This graphic outlines the reintroduction program in the Wenatchee River. Reintroduction was started by releasing lower Columbia River (LCR) smolts beginning in 1997. Returning two‐ to three‐year‐old fish (middle Columbia River; MCR) were used as broodstock in subsequent years with some reliance on LCR stock in generation 1. Numbers in parentheses indicate the successfully genotyped fish for each broodyear. The A, B, and C broodlines identified by principal coordinate analysis are denoted

During the initial reintroduction (1999–2001), juvenile smolts raised in lower river hatcheries were released into the Wenatchee River. In later years, adult fish returning to the Wenatchee River were used to produce locally acclimated juvenile Coho salmon. Tissue samples (fin clips) were collected from these adult broodstock from 2000 to 2011 for DNA extraction and genotyping. Supplementation with lower river stocks was used in 2000 and 2002 but in subsequent years adult fish returning to the Wenatchee River system became suitably abundant to meet hatchery production and escapement goals (Figure 1). A total of 664 samples were included for genotyping, with four generations of fish represented from each of three broodlines (Figure 1). However, samples were not available from one generation (year 2003) of broodline “A.” Broodline groups were labeled “A” (2000, 2006, and 2009), “B” (2001, 2004, 2007, and 2010), and “C” (2002, 2005, 2008, and 2011) and will be referred to as such hereafter (Figure 1).

### 2.2 Library preparation and genotyping

Genomic DNA was extracted from fin tissue using Qiagen DNeasy 96 kits and quantified with a Tecan m200 96‐well plate reader fluorimeter using Invitrogen Quant‐iT^™^ pico green reagent. The DNA concentration of each sample was determined by the standard curve method using samples of known DNA concentration. Restriction site‐associated DNA (RAD‐seq; Miller et al. 2007) libraries were prepared by first digesting 500 ng of each sample with SbfI‐HF (NEB; New England Biolabs, Ipswich, MA, USA) followed by heat inactivation of the enzyme. The remainder of the preparation followed the instructions of previously published studies (Hecht, Campbell, Holecek, & Narum, 2013; Miller et al., 2012). In brief, adapters containing six base barcodes were ligated onto the *SbfI* cut sites using T4 ligase (NEB). Uniquely barcoded samples were then combined into pools of 48 individual samples per pool for further preparation. Three hundred microliters of each DNA pool was sheared to an average size range of 500 base pairs using a Bioruptor sonicator (Diagenode). The sheared DNA was then purified using Qiagen MinElute columns, size selected using Agencourt Ampure XP magnetic beads to a size range of 200 to 700 base pairs, blunt‐ended using a quick blunting kit (NEB), A‐tailed using Klenow fragment DNA polymerase (NEB), second adapter added using T4 ligase (NEB), amplified with 14 cycles of PCR using Phusion Master Mix (NEB), and purified with Agencourt Ampure XP beads. Final library concentrations were quantified using 2× Sybr Green Master Mix on a QuantStudio 6 (Thermo Fisher) qPCR instrument and normalized to a concentration of 5 ng/μl. Each library was then sequenced with an Illumina HiSeq 1500 instrument for 100 cycles from the read 1 primer site (single read). A single library was chosen to run as a paired‐end run with 100 cycles in each direction to create longer contigs for individual loci later.

Genotypes were generated from the raw sequencing data using the de novo pipeline in the program Stacks v1.03 (Catchen, Hohenlohe, Bassham, Amores, & Cresko, 2013). Raw sequences were trimmed to 80 bases, quality filtered, and binned into fastq files for each individual sample using the “process_radtags” script. In samples where an insufficient number of sequencing reads were collected, additional libraries were created to attempt to collect enough sequencing data for adequate genotyping. Where applicable, sequencing reads from the same individual (but different libraries) were concatenated. Individual samples with less than 1M total reads were immediately excluded from the dataset. The average number of raw reads for the remaining samples was 2.9M but varied greatly among samples (1M–14M). As higher numbers of raw reads increase the likelihood that reads with convergent sequencing errors will be combined into a stack, the minimum stack depth (m parameter) used with the “ustacks” program was scaled according to raw read numbers. The m parameter was set to the nearest integer using the formula (raw reads in millions * 2). The distance allowed between stacks (M parameter) was set at 2. The cstacks portion of the pipeline to build the catalog of SNPs included a total of 22 ascertainment samples, with two individual samples from each year class of the three broodlines that had between 2.5M and 3.5M reads per sample. The number of mismatches allowed between sample tags when building the catalog was set to 2 (n parameter). The sstacks module was then run with all samples using the newly created catalog, and the output data were then compiled into a raw vcf (variant call format) file using the “populations” module (rather than outputting to a mysql database). SNP loci were then filtered directly from the vcf output using a custom perl script which enforced a minor allele frequency cutoff of 5.0%. Samples with less than 75% of the genotypes from the remaining SNP loci were then removed from the dataset followed by excluding any remaining SNP loci with less than 80% of the genotypes within the remaining samples. In order to account for SNP loci with potential null alleles and paralogous sequent variations (PSVs), deviation from Hardy–Weinberg expectations was then tested for all remaining loci using all the remaining samples as a single population. Loci with corrected *p*‐values less than the BY‐FDR (Benjamini & Yekutieli False Discovery Rate; Benjamini & Yekutieli, 2001) corrected value (α = 0.01; FDR corrected *p*‐value = 1.8 × 10^−6^) were removed from the dataset.

### 2.3 Testing for SNP loci under selection

The remaining samples and loci were converted to GenePop files and imported into Excel using the GenAlEx plug‐in (Peakall & Smouse, 2006). The collections from each year were used as independent populations for the purposes of identifying possible underlying genetic structure. A principal components analysis (PCA) was conducted using pairwise genetic distance data generated using GenAlEx. Each of the three broodlines was treated as biological replicates of the same reintroduction experiment. Genotype data were split into three datasets with each broodline composed of four generations of adult broodstock fish.

In order to protect against false positives in outlier tests (Lotterhos & Whitlock, 2014; Narum & Hess, 2011), three different methods were used to identify potential candidates for divergent selection within each broodline. As each broodline was tested separately, there was minimal underlying population structure that could bias outlier results. The methods included linear regression of allele frequency changes, and two standard applications of outlier tests implemented with software programs Lositan (Antao, Lopes, Lopes, Beja‐Pereira, & Luikart, 2008), and Bayescan 2.0 (Foll & Gaggiotti, 2008). In order for loci to be considered candidates under divergent selection in our final outlier results, they had to be significant in at least two of the three methods as applied below.

A linear regression method was used as an initial test for loci with significant changes in allele frequency across generations within each broodline. To distinguish potential loci under selection from those experiencing random genetic drift, a significance threshold was established to identify SNPs with a greater change in allele frequency than expected by chance for neutral markers. Rather than simply applying the frequentist approach of classifying all slopes outside of an arbitrary cutoff value as outliers (i.e., two standard deviations) which will always return approximately 5% of the loci, we applied a different strategy which would allow the possibility that no loci were significant. To identify outliers that were considered statistically significant from neutral loci, a Q–Q test was performed using the slopes for each locus for each of the broodlines. A chi‐square test was performed for each locus using the difference between the expected slope (based on Q–Q linearity) and observed slope at each of the loci. Loci with *p*‐values of less than .01 were designated as outliers. This cutoff value corresponded to a deviation from Q–Q linearity of 3.56× the average deviation across all loci.

A second test for loci under selection utilized the program Lositan (Antao et al., 2008). This program examines the relationship between *F*_ST_ and heterozygosity (*H*_e_) at each locus and identifies loci outside of the specified confidence interval for neutral loci as outliers. This program was executed separately for each of the three broodline datasets specifying samples from each generation as a population using the “infinite alleles” mutation model. The confidence interval was set to 0.95, the false discovery rate was set to 0.1, the subsample size was set to 50, the number of simulations was set to 50,000, and the program was set to first calculate a neutral mean *F*_ST_. Samples from the 2007 year class were excluded from the analysis due to small sample size (*N *=* *7).

The program Bayescan was used as a third approach to identify loci under selection within the datasets (Foll & Gaggiotti, 2008). For these analyses, the 2007 data were not omitted due to the reported lack of bias with small sample sizes when using this method. A custom perl script was used to convert the GenePop formatted genotype files for each of the three broodlines to Bayescan formatted input files. The program was run using default parameters using the SNP genotypes as regular codominant data.

The locations of all RAD loci used in the analyses were mapped to the Okis_V1 reference genome assembly (GenBank assembly accession: GCA_002021735.1). As the traditional RAD library preparation method uses sonication to randomly shear the DNA following P1 adapter ligation, the resulting library constructs are of varying length. Some of the R2 reads will overlap with the R1 reads allowing their assembly into longer contigs. Using the RAD tag sequences for each locus, we used a series of custom perl scripts to sample paired‐end data for assembly into longer contigs using the CAP3 program (Huang & Madan, 1999). The program bowtie2 (Langmead & Salzberg, 2012) was used to align RAD sequences to the two reference genomes in order to determine the most likely genome locations of each of the outlier loci. Genome positions with a mapQ score of less than 10 were discarded and when a locus mapped to multiple positions, all possible locations were retained. Another custom perl script was used to identify the three nearest genes within 100,000 bases of the mapped outlier loci using the annotation file for the reference assembly.

## 3 RESULTS

Tissue samples collected over the course of the reintroduction period varied greatly in quality and many samples failed to produce genotype data meeting the minimum requirements for inclusion in the final dataset. Poor genotyping was generally the result of low numbers of sequencing reads for many of the individual samples. After the initial screening of 12 libraries containing between 81 and 96 individuals each, 192 samples were targeted for additional sequencing (two HiSeq lanes) in order to boost the total number of raw sequencing reads to the target range of 2–4M reads. However, only 68% of the resequenced samples produced enough combined reads for the genotypes to pass filtering. In total, 664 samples were utilized for the analyses of the 1,103 attempted for RAD genotyping after quality filtering of the genotype data. Quality filtering of the SNP loci identified using the denovo Stacks pipeline left a total of 5,392 markers in the dataset.

**Figure 2:**
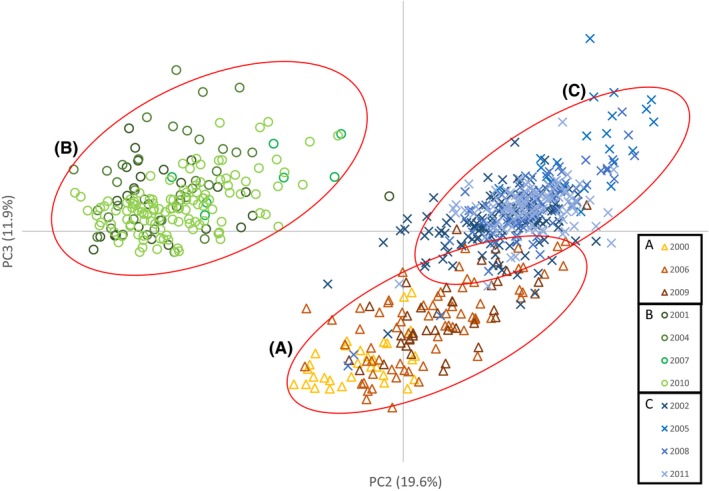
Principal coordinate analysis reveals genetic distinctions between brood years in reintroduced Wenatchee River Coho salmon. These clusters were termed the A, B, and C broodlines

Temporal genetic structure among the three broodlines of reintroduced fish was evident from PCA plots that revealed three distinct clusters (Figure 2). Each cluster corresponded to a broodline from one of the initial return years (2000, 2001, and 2002) and their descendants. This was likely the result of genetic drift between year classes within source stocks due to a lack of intergenerational gene flow (i.e., spawning is limited to the 3‐year‐old age class) as has been observed previously in hatchery‐reared stocks of Coho salmon (Smith et al., 2015). Broodline “B” was the most distinct from the other two broodlines in the PCA plot (Figure 2). As each of these lineages was genetically distinct, each was treated as a separate dataset for remaining analyses. Each dataset was composed of the initial founder stock and 2–3 generations of descendants (no samples were collected in brood year 2003).

**Figure 3:**
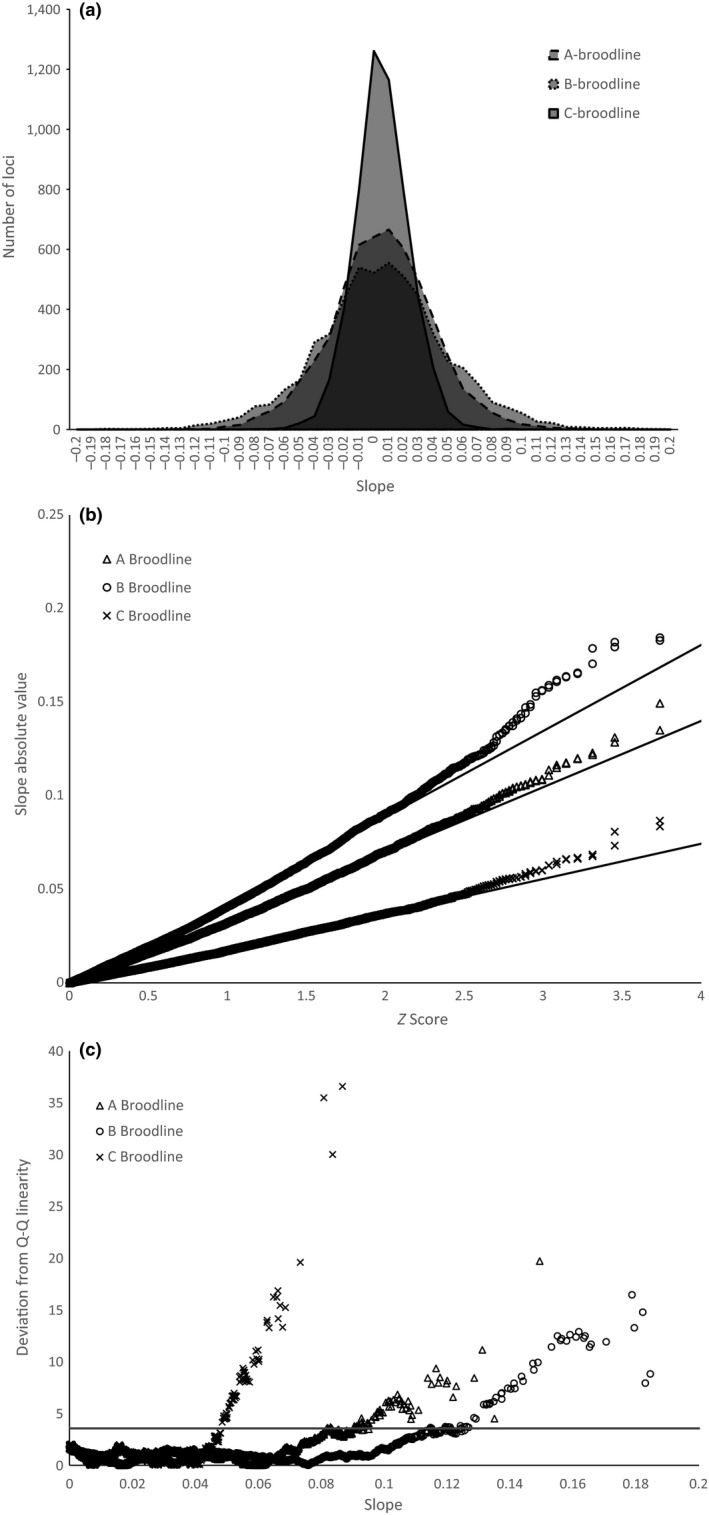
Histogram of slopes derived from linear regression of allele frequency differences at each of the 5,392 SNP loci over four generations of reintroduced fish (a). A Q–Q plot showing the expected linear distribution of slopes given a normal distribution for the number of data points observed and the deviation from linearity on the tails of the distribution for each of the three broodlines (b). For each of the observed data points, the deviation from the Q–Q plot linearity was calculated and plotted against the slope (c). The cutoff line in the figure was set to a significance threshold determined by chi‐square test (threshold equivalent to a *p*‐value of .01)

The first method used for detecting loci under selection was to inspect the genotype data for significant changes in allele frequency over time. Analysis of allele frequency trends showed a normal distribution of slopes within each of the three broodline datasets (Figure 3a). Broodlines A and B, however, showed a broader distribution that is likely reflective of lower accuracy in the allele frequency estimates in those datasets due to low representation within some brood years (Figure 1). Loci under selection were expected to have the steepest slopes compared to those under random genetic drift as reflected by the linearity of ordered loci in Q–Q plots (Figure 3b). Deviation from linearity at the posterior end of the distribution was observed for each broodline which revealed loci with slopes that were greater than would be expected for normally distributed data (Figure 3b). Chi‐square analysis was used to identify a significance cutoff (α = 0.01) of 3.56× the average deviation from linearity for each dataset (Figure 3c). A total of 169 loci were identified as significant outliers using this method, and nine outlier loci were observed in common among two broodlines. No loci were identified as outliers in all three broodlines using this method (Data S4).

**Figure 4:**
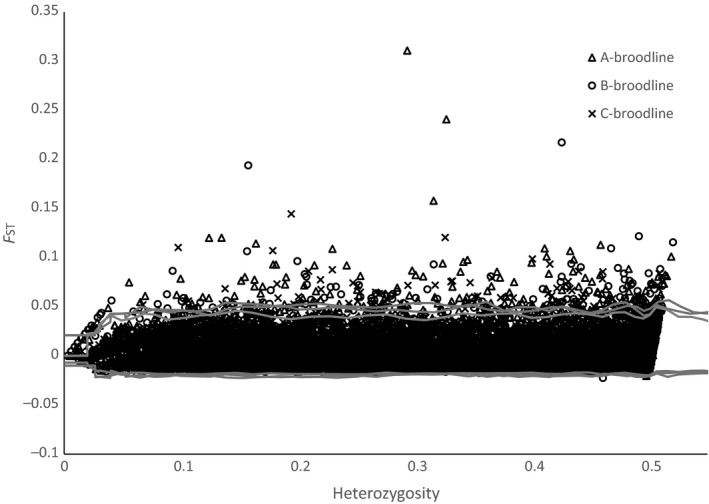
*F*_ST_ outlier analysis plot using Lositan. The gray lines represent the 95% confidence interval for putatively neutral loci for each of the three broodlines analyzed

The program Lositan identified 179 loci as being significant candidates for directional selection (Figure 4). Of the 179 significant loci, six were identified in two broodlines (Coho_64855‐71, Coho_87190‐34, Coho_69193‐61, Coho_27527‐68, Coho_68125‐68, Coho_115799‐69), and a single locus (Coho_92766‐45) was identified as an outlier in all three broodlines (Data S4).

**Figure 5:**
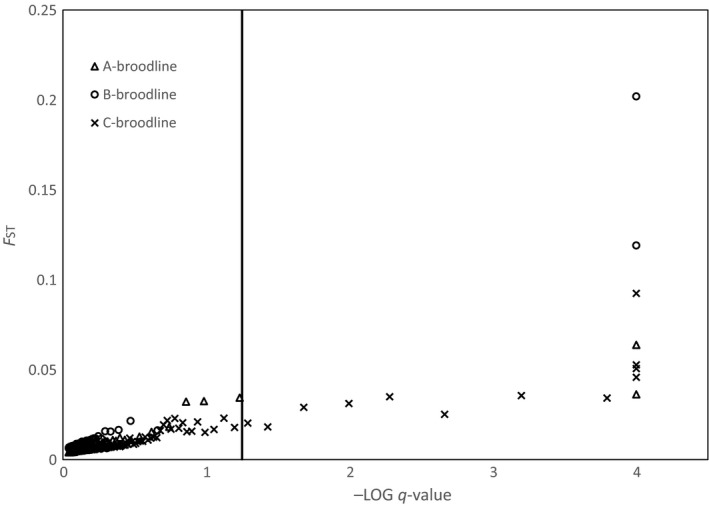
Bayescan analysis plot showing outlier loci for each of the three broodlines

**Figure 6:**
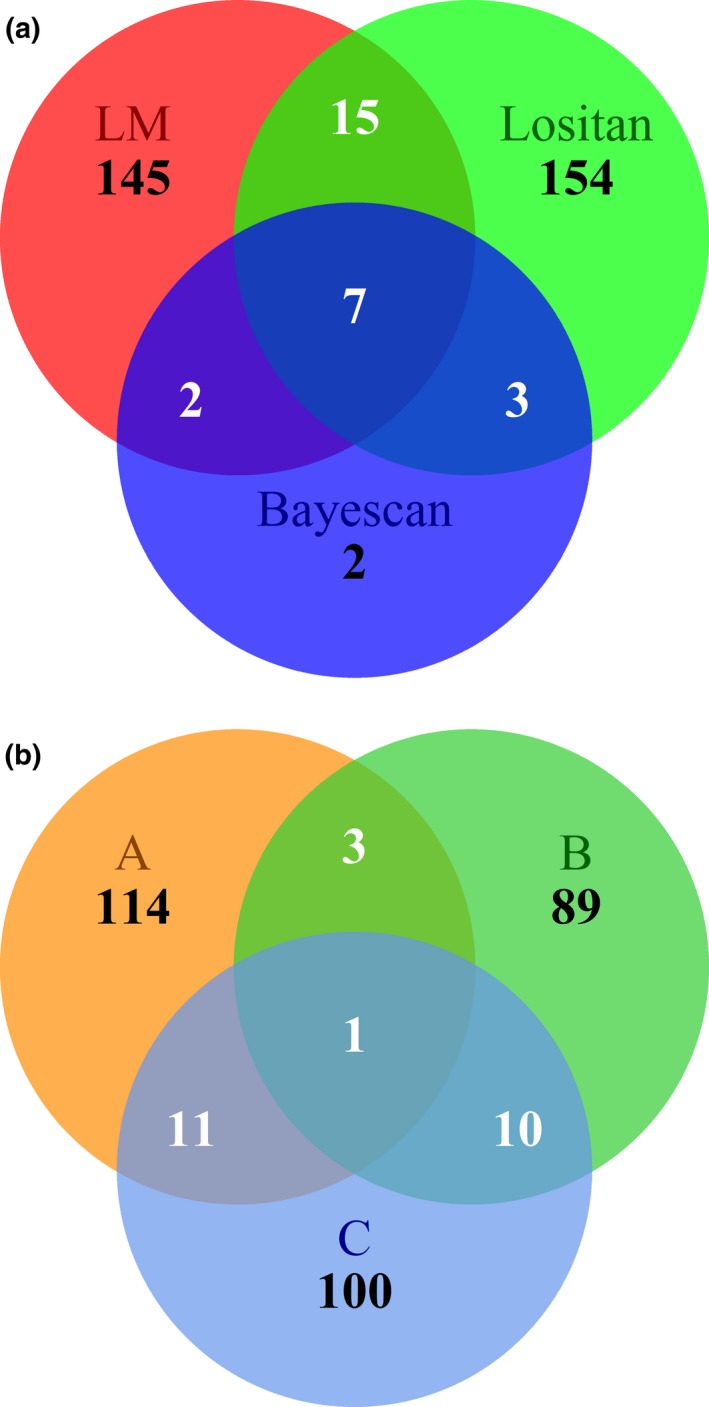
Venn diagram indicating loci identified as being under selection using the three analysis methods (a). Loci identified within and between the three broodlines are illustrated in the second Venn diagram (b)

Results from Bayescan identified a total of 14 loci as being under selection (Figure 5), with two loci (Coho_64855‐71 and Coho_87190‐34) identified in two broodlines (Data S4). No loci were identified as outliers in all three broodlines. All but two loci identified using this analysis method were also identified as significant with one or both of the other methods used to detect loci under selection (Figure 6a). Across outlier methods, a total of 25 loci were in common in two broodlines, and one marker in all three broodlines (Figure 6b).

**Figure 7:**
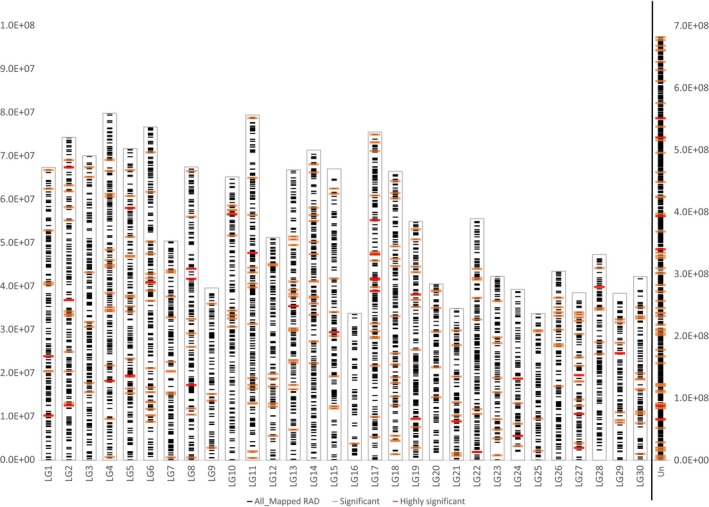
Illustrates the genome positions of 5,009 of the 5,392 RAD loci used in the study (small black bands). Significant and highly significant loci are indicated by the larger orange and red bands, respectively. The 21,216 unplaced scaffolds represent approximately 27% of the total genome size and are therefore presented on a secondary axis on the far right

Extended consensus sequences of 5,009 of the 5,392 RAD loci mapped to at least one location within the Okis_V1 genome assembly (Data S5 and S6). Sequences of the 328 candidate loci identified as being under selective pressure by at least one of the nine outlier analyses (three broodlines x three analysis methods) were aligned to the *O. kisutch* (Okis_V1) reference genome. Of the 37 highly significant candidate loci (significant in at least two of the outlier tests), 31 mapped to at least one location within the *O. kisutch* genome. No significant clustering of the mapped loci was observed within specific regions of the genome (Figure 7). The three nearest genes within a window of 100,000 bases of the genome locations of the 31 highly significant RAD tags were identified and 25 of these loci returned 108 nearby genes. These nearby genes, their genomic location, and their distances from the marker were compiled into a table and are included as supplemental information (Data S3). The most significant marker mapping to a single location in the genome assembly was Coho_64855‐71 which lies within intron 6 of the sodium channel and clathrin linker 1‐like gene (four reported isoforms: XP_020324990.1—XP_020324993.1). This gene resides within an unanchored scaffold (MPKV01000936.1—54,120 bases) which contains no other RAD loci.

## 4 DISCUSSION

This study provided an opportunity to evaluate neutral and adaptive genetic variation in Coho salmon throughout the process of colonization to a new habitat following reintroduction from source stocks. Genetic analyses revealed that reintroduction of Coho salmon in the Wenatchee River began with three genetically distinct broodlines. Through the first adult returns and the subsequent three generations, there was evidence of limited gene flow between these stocks. Throughout their natural range, Coho salmon typically have overlapping generation times as adults return to spawn primarily as three year olds, but two‐year‐old jacks (early maturing males) are also common and provide gene flow across generations (Beacham, Wetklo, Deng, & MacConnachie, 2011; Sandercock, 1991). However, hatchery programs of Coho salmon often limit the use of jacks within broodstock and distinct broodlines that follow three‐year intervals can develop (Conrad, Gilbert‐horvath, & Carlos, 2013; Smith et al., 2015). This phenomenon of distinct temporal structure between broodlines was apparent at the initiation of the reintroduction program in the Wenatchee River and reflects spawning practices from source hatcheries used for reintroduction. However, distinct temporal structure persisted among broodlines in the Wenatchee River over subsequent generations despite increased potential for gene flow from jacks through natural spawning and inclusion in the local broodstock program. Broodline “B” was the most distinct from the other two broodlines and may have trait characteristics (e.g., differential run‐timing) that maintains isolation among broodlines as has been observed in other studies of this species (Smith et al., 2015). However, run‐timing and other trait characteristics were not available for individuals in this study.

The three distinct Coho salmon broodlines in this system provided the opportunity to examine biological replicates for signals of local adaptation over four generations and this paradigm was the basis for our analysis strategy for detecting SNP loci under selection following reintroduction. In total, 37 loci were identified as significant outliers in at least two of the nine tests and were considered the most likely candidate loci, leaving another 291 loci identified as significant in only a single test. While mapping these likely candidate loci revealed many genes in this list were involved in processes that are potentially beneficial for adaptation to a long‐distance migration, the lack of a significant genomic “hotspot” and the low density of SNP markers used in this study was not sufficient to speculate which nearby genes are responsible for the adaptive signal. Similarly, RAD sequencing is a reduced representation approach for genome scans and therefore may miss regions of adaptive variation in species with rapid linkage decay and small linkage blocks (Catchen et al., 2017; Lowry et al., 2017; McKinney, Larson, Seeb, & Seeb, 2017; Tiffin & Ross‐Ibarra, 2014). Therefore, we interpret these candidate loci cautiously until further studies with more extensive genomic coverage can more confidently map genetic adaptation. The most significant RAD locus within our dataset (Coho_64855‐71) mapped within an intron of a sodium channel gene suggesting variations within this gene may be important for long‐distance migration. However, for the reasons described above, this result will require further validation.

In this study, we utilized multiple tools developed for detection of adaptive loci between populations in order to reduce false positives in our dataset. Specifically, we required outliers to be based on replicates and overlapping results among methods in order to be considered as candidate loci (François, Martins, Caye, & Schoville, 2016; Lotterhos & Whitlock, 2015). This approach proved necessary as each outlier method that we implemented included caveats that could have led to elevated signals of false positives if interpreted alone. The linear method for detection of loci under selection relies on the accuracy of the allele frequency estimates at each generation in order to provide accurate data points. In some year classes, only a few individual samples remained after quality filtering, and allele frequency estimates would be expected to be less accurate. A reflection of this inaccuracy could manifest in the range of slopes seen in each of the broodlines with respect to their sample sizes. Although each broodline showed a range of slopes with a normal distribution centering on zero, a broad range of slopes was observed in the “A” and “B” broodlines when compared to the “C” broodline which contained the most samples per generation. Likewise, the “A” and “B” broodlines showed higher degrees of deviation from linearity in the Q–Q plots. This could also be an indication of higher degrees of genetic drift within the “A” and “B” broodlines manifesting as generalized genome‐wide changes in allele frequency. However, the pattern of higher slope ranges with lower sample numbers indicates that this is more likely the result of inaccurate allele frequency estimates in year classes with small sample numbers. Although there is more statistical noise in the “A” and “B” broodline datasets, outlier loci still deviated significantly from expectations and many of the outlier loci were also identified as significant using the other analysis methods.

Similar to the linear model, Lositan is also sensitive to small sample sizes due to inaccuracies in allele frequencies used to calculate *F*_ST_ and heterozygosity. For the “B” broodline analysis, the 2007 samples (*N *=* *7) were removed due to unrealistically high *F*_ST_ estimates when included. As *F*_ST_ is a measure of genetic distance between collections (usually populations) and our collections are different year classes of the same population, the average *F*_ST_ should be very low. This is indeed what is observed in the data with only a few outliers exceeding the 95% confidence interval for neutral loci. Nevertheless, disproportionately high *F*_ST_ may be reported at some loci due to small sample numbers and possibly low genotyping percentage within one or more collections resulting in false positives. The false‐positive rate for this method may even exceed that of the linear method as inaccuracies at a single collection would have a greater effect on the analysis than would a single erroneous data point used in linear regression.

Unlike the other two methods used for detection of SNP loci under selection, Bayescan incorporates uncertainties in allele frequency estimation based on sample size and often has less false positives than other outlier tests (De Mita et al., 2013; Lotterhos & Whitlock, 2014; Narum & Hess, 2011). Not surprisingly, Bayescan also returned the fewest number of SNP loci under selection (*N *=* *16). However, of these loci, only two had not also been identified using the linear method or Lositan revealing agreement between the analysis methods.

In conclusion, our study identified patterns of neutral variation that reflected degrees of reproductive isolation among three broodlines that have been perpetuated from source stocks. While these broodlines have increased opportunity for gene flow in nature from overlapping age classes (Smith et al., 2015), it is uncertain how much time will be necessary to reduce or eliminate temporal variation in this system. Additionally, patterns of adaptive variation indicate many candidate loci with signals of divergent selection following reintroduction of Coho salmon to new spawning habitat. This genome scan for adaptive loci was independent of a‐priori selection of candidate markers or association with phenotypic traits. Tracking genetic variation over four generations following reintroduction to a new habitat revealed a total of 328 unique loci that showed a significant signal, 26 were identified as likely outliers due to consistent results in at least two of the broodlines, and 27 were positive in two or more analysis methods, lending some credibility that the identified loci are not simply noise (i.e., false positives). Although the marker density was insufficient to confidently implicate specific adaptive genes, a single potentially adaptive gene is reported. These data indicate that the genomes of these reintroduced Coho salmon are changing in response to their new spawning habitat and increased migration distance.

## CONFLICT OF INTEREST

None declared.

## AUTHOR CONTRIBUTIONS

NRC analyzed the sequencing data, conducted the statistical analyses, and wrote the manuscript. CK is the lead biologist overseeing the reintroduction of Coho salmon in the middle Columbia River, he coordinated the collection of the samples, consulted on the study design, and edited the manuscript. KM is also a lead biologist with Yakama Nation fisheries implementing the Coho reintroduction strategy in the Wenatchee River and edited the manuscript. SRN designed the study, oversaw the analysis of the data, and edited the manuscript.

## DATA AVAILABILITY

Raw sequencing data are available in the NCBI sra database (Accession number: BioProject Accession numbers PRJNA141569 and PRJNA414579 – in process will be made available upon acceptance). Supplemental data include RAD tags with SNP loci, SNP genotypes, annotations of genes near significant SNPs, outlier test results, extended consensus sequences from RAD loci used in the study, and sam formatted alignment file with genome locations for RAD loci mapped to the Okis_V1 genome.
